# Distribution of birds in Colombia

**DOI:** 10.3897/BDJ.9.e59202

**Published:** 2021-02-03

**Authors:** Danny Vélez, Edwin Tamayo, Fernando Ayerbe-Quiñones, Julián Torres, Juan Rey, Carolina Castro-Moreno, Bryan Ramírez, Jose Manuel Ochoa-Quintero

**Affiliations:** 1 Universidade Federal do Rio de Janeiro (UFRJ), Museu Nacional (MN), Departamento de Entomologia, Laboratório de Hymenoptera, Rio de Janeiro, Brazil Universidade Federal do Rio de Janeiro (UFRJ), Museu Nacional (MN), Departamento de Entomologia, Laboratório de Hymenoptera Rio de Janeiro Brazil; 2 Instituto de Investigación de Recursos Biológicos Alexander von Humboldt, Bogotá, Colombia Instituto de Investigación de Recursos Biológicos Alexander von Humboldt Bogotá Colombia; 3 Wildlife Conservation Society, Colombian Program, Popayán, Colombia Wildlife Conservation Society, Colombian Program Popayán Colombia

**Keywords:** Aves, biodiversity, conservation, data mobilisation, decision-making, GIS, sustainable use

## Abstract

**Background:**

1. Colombia with 1941 known recorded bird species is one of the most species rich countries in the world. Efforts are necessary to conserve, study and promote sustainable use of this important taxonomic group throughout Colombia’s vast territory.

2. In an ideal world, informed decisions that are based on sound scientific information should be likelier to have successful outcomes. Nevertheless, there are barriers that make it difficult to access and use information in a timely fashion. Those same barriers impede the study, conservation and sustainable use of bird species in Colombia. On the other hand, given that there is good documentation about the ecology of a large number of species, information about the distribution of birds can be easily incorporated into decision-making processes, once this information becomes readily available in a consumable format using Geographic Information Sciences tools.

3. In this context, the main objective of this paper is to present the first compilation of the current distribution of 1889 (97%) species of birds in Colombia, using expert criteria. The shapefiles were used to show the distribution and diversity of bird species in Colombia under both geopolitical and conservation geographic units.

4. The information provided in this paper can be used as a baseline for a huge number of initiatives that aim to strengthen conservation efforts and improve knowledge about one the most unique taxonomic groups in the country. These range from land use planning strategies at the municipal or department scale to sustainable use of bird species - such as those initiatives related to bird watching - in Colombia.

**New information:**

This study has considered three key aspects: 1) the importance of birds for Colombia’s ecosystems, 2) the privileged place of Colombia in bird species richness and 3) the importance of data mobilisation in formats easily consumable by Geographic Information Systems (GIS) to facilitate the processes of informed decision-making. We present the first compilation - in shapefile format - for 1889 of the 1941 bird species recorded from Colombia. Using this novel collection, we showed the species richness of birds in Colombia’s 33 Departments plus its Captial District (DPs), 1122 Municipalities (MNs), 58 protected areas (PAs), 39 Regional Autonomous Corporations (the authorities responsible within their respective jurisdictions for regulating the environment and renewable natural resources in Colombia; CARs) and 916 Collectively Titled Territories (including both indigenous reservations and afro-descendant communities; CTTs). In addition, we provide a list of known bird species richness for the above geographic units found in the available literature. The information provided here can be used as a baseline for a huge number of initiatives concerning the study, conservation and sustainable use of bird species present in Colombia, providing access to key features of bird distribution that should facilitate decision-making.

## Introduction

Birds inhabit almost every ecosystem on earth and are amongst the most diverse, active and important ecosystem service providing groups (Sekercioglu 2006). Given the large body of knowledge about birds, they are frequently used in initiatives related to conservation and sustainable use of biodiversity around the world (Hausmann et al. 2019). Colombia, with 1941 known bird species (Ayerbe-Quiñones 2019), is amongst the countries with the highest species richness of this biological group worldwide. Given the above, special effort is required to make information about Colombian birds freely available for research, conservation and sustainable use management and planning.

In an ideal world, decisions that are informed by sound data should have better odds of producing successful outcomes. Actions related to the study, protection and sustainable use of birds must be supported by information about the species and their relationships with both biotic and abiotic components of ecosystems. Activities that require data on bird distributions include: national planning and budgets for resource management in sectors such as agriculture, mining, infrastructure, protected areas, compliance with multilateral environmental agreements; development of environmental resource legislation; measurement and mitigation of human impact on the environment; mitigation of anthropogenic drivers and conflicts; and projects on sustainable use of biodiversity (Stephenson et al. 2017). Nevertheless, there are barriers that make it difficult for consumers to access and use information in a timely fashion.

Stephenson et al. (2017) pointed out several barriers that make it difficult to access and use information for biodiversity management. The four groups of barriers identified in the aforementioned paper are: 1) data availability, 2) data quality and usability, 3) willingness to collect and use data and 4) technical and financial capacity. However, with both constant technological advances and increasing necessity for information, an important ecosystem of tools, institutions and initiatives has grown with the aim of increasing technical capacity in biodiversity data mobilisation, use and reuse. Nevertheless, there are key issues remaining to be addressed that concern both data and domain integration of biodiversity information (König et al. 2019).

Data and information are valued in the degree in which they are findable, usable and in formats consumable by Geographic Information Sciences (GIScience), relevant to decision-making processes. Foody (2008) concluded that GIScience provides both data on environmental properties and techniques to explore, visualise, use and integrate geographic information with other data (e.g. biological data) for understanding biodiversity and conservation. For these reasons, we were inspired to create an invaluable information resource that would be useful as baseline to understand and conserve bird species based on their geographic distribution in Colombia.

## General description

### Purpose

Given the importance of data mobilisation for democratising information about birds in Colombia, the main objective of this paper is to present a shapefile with the current distribution of 1890 species of birds recorded from Colombia and use the obtained distribution to show some key features of bird species richness in Colombia in both geopolitical and conservation geographic units to facilitate decision-making processes, scientific research and sustainable use of biodiversity in Colombia.

## Sampling methods

### Step description

We used 1889 expert-based bird distribution maps obtained from the Guide to Birds of Colombia (Ayerbe-Quiñones 2018). The bird distribution maps in PNG format were georeferenced and transformed into a raster format and posteriorly into a vector shapefile format using the Python Programming Language 2.7 and the software ArcGIS 10.7. Once we had the individual bird species maps in shapefile format, we used this information to obtain some key features and figures on bird species richness in Colombia. To perform this task, we evaluated distributions in three key management units in the country: geopolitical units, management and conservation units and collective territories.

To obtain the bird species number per geopolitical, natural, management and collectively titled territories, we made layer intersections between the shapefile of bird distribution and the layers for each one of the selected territories, using the EPSG 4686 system in the software ArcGIS v. 10.7 software. Layers used for intersection were: DPs and MNs (IGAC 2019), PAs (PNNC 2019), CARs (IDEAM 2018) and CTTs (Etnoterritorios 2020a, Etnoterritorios 2020b). Metadata for each one of the layers can be consulted in the respective data resource.

Finally, to make the compilation freely available, we uploaded it into the online data repository of the Instituto de Investigación de Recursos Biológicos Alexander von Humboldt (http://geonetwork.humboldt.org.co/geonetwork/srv/spa/catalog.search#/home/). In addition, the distribution map of each species was uploaded into the BioModelos platform where it can be consulted by species name (http://biomodelos.humboldt.org.co/).

## Geographic coverage

### Description

To establish the geographic coverage, we used the EPSG 4686 coordinates system. All the Colombian territory is included in this work.

### Coordinates

-4.204 and 13.390 Latitude; -81.763 and -66.829 Longitude.

## Taxonomic coverage

### Description

In this paper, we follow the taxonomic system of Ayerbe-Quiñones (2019).

### Taxa included

**Table:** 

Rank	Scientific Name	Common Name
class	Aves	Birds

## Usage licence

### Usage licence

Other

### IP rights notes

Creative Commons Attribution (CC-BY) 4.0 License.

## Data resources

### Data package title

Distribution of birds in Colombia. Year 2020

### Resource link

http://geonetwork.humboldt.org.co/geonetwork/srv/spa/catalog.search#/metadata/5c2b19d2-6893-4955-aa65-509d1c3f2706

### Number of data sets

2

### Data set 1.

#### Data set name

BIRD_Colombia

#### Data format

Shapefile

#### Number of columns

3

#### Download URL

http://geonetwork.humboldt.org.co/geonetwork/srv/api/records/5c2b19d2-6893-4955-aa65-509d1c3f2706/attachments/BIRD_Colombia.rar

#### Description

The .Zip file contains a shapefile with the distribution of bird species in Colombia. The shapefile has three attributes.

**Data set 1.** 

Column label	Column description
Order	Scientific name of the Order in which the species is classified
Family	Scientific name of the Family in which the species is classified
Species	Bird species

### Data set 2.

#### Data set name

Additional data

#### Data format

EXCEL file

#### Number of columns

7

#### Download URL

http://geonetwork.humboldt.org.co/geonetwork/srv/api/records/5c2b19d2-6893-4955-aa65-509d1c3f2706/attachments/BIRD_Colombia.rar

#### Data format version

.xlsx

#### Description

Within the same .Zip that contains the BIRD_Colombia shapefile, there is an Excel file with three sheets where the number of bird species per Municipalities, Indigenous Reservations and afro-descendant communities is shown. The Excel file has seven attributes.

**Data set 2.** 

Column label	Column description
Code_Dane	MN unique identifier assigned by the Colombian “*Departamento Administrativo Nacional de Estadística*” (DANE)
Department	Name of the Department
Municipality	Name of the Municipality
Spp	Number of species
Indigenous reservation name	Name of the reservation
Etnia	Name of indigenous ethnicity
Afro-descendant community	Name of the community

## Additional information

As pointed out by Chapman (2005) - referring to primary species-occurrence data - biodiversity information has endless uses in almost every aspect of human endeavour worldwide. These include aspects that are not so obvious, such as food security, education and recreation. The same author called attention to the fact that it is necessary to make maximum use of these data to better understand biodiversity, to mitigate and monitor changes to our environment and to improve, conserve and sustainably use our biodiversity. In this context, the Shapefile compilation presented here has the potential to facilitate and improve research, conservation and sustainable use of biodiversity in Colombia, with a special focus on the country’s charismatic and ecologically-important birdlife.

Expert-based range maps for species distribution - like those presented here - are useful tools that help in research and conservation of biodiversity. With some limitations, these kinds of distributional models, based on group expert criteria, become valuable in cases in which there is a lack of reliable information on the distribution of biological species (e.g. Fourcade 2016, Mainali et al. 2020) and as a complement to both species distribution and ecological niche modelling techniques (e.g. Fourcade et al. 2013, Merow et al. 2017). Such maps can help to establish an information baseline to understand biodiversity patterns in poorly-known species. We consider that the lack of information for several species of birds in Colombia can be remedied in the meantime by the models presented here, which can be used as a starting point for many research, conservation and decision-making processes. A dynamic process for improving information on species distributions in one the most biodiverse countries of the world will make it possible to generate a Living Atlas of Colombian Biodiversity that can be improved over time with the help of experts and the inclusion citizen science data.

Key figures on bird species richness in Colombia per different geographic units are presented in Tables 1, 2, 3, 4, 5. Information provided in these Tables can be used for a quick comparison between the number of species in different geographic units. Here, we present diverse examples of the information and comparisons that can be made in different territories. Additionally, we provide a comparison to available information - including both eBird (2020) database and literature - of the number of species recorded per DPs and PAs (Tables 1, 4). The differences amongst the number of species presented by sources are due to differences in the methods and sampling effort used to obtain the information. Expert maps use a coarser resolution than other methods; this can explain the tendency to obtain the highest number of species per geographic unit with this method. As noted above, expert maps are used as a baseline and complement knowledge that can be used for education, decision-making processes and research.

## Figures and Tables

**Table 1.** Number of bird species per DPs in Colombia obtained in this paper and compared with available references, including eBird (2020).

**DP**	**Number of bird species this work**	**Number of bird species eBird July 2020**	**Number of bird species in other sources**	**References of other sources**
Cauca	1409	1164	1102	Ayerbe-Quiñones et al. 2008
Nariño	1384	964	1048	Calderón-Leytón et al. 2011
Antioquia	1125	1047		
Boyacá	1107	913		
Meta	1063	1048		
Cundinamarca	1062	919	941	Chaparro-Herrera et al. 2018
Chocó	1059	941		
Putumayo	1050	1000		
Caquetá	1046	850		
Valle del cauca	982	1024	989	Cárdenas et al. 2020
Norte de Santander	940	654		
Córdoba	929	632	504	Ballesteros et al. 2015
Cesar	882	613		
Santander	874	844		
Caldas	861	883	923	Corporación Autónoma Regional de Caldas and Asociación Calidris 2010
Casanare	858	688	507	Zamudio et al. 2011
Tolima	822	794		
Arauca	798	554	512	Izquierdo et al. 2019
La guajira	770	581		
Huila	748	765		
Bolívar	728	598		
Risaralda	719	894		
Magdalena	697	647		
Amazonas	690	639		
Guaviare	678	579		
Vichada	639	559		
Quindío	633	687		
Guainía	614	546		
Sucre	584	405		
Vaupés	572	610	558	Carrillo et al. 2018
Atlántico	510	403	363	Castro-Vásquez 2016
Bogotá, D.C.	489	535		
Archipiélago de san Andrés, Providencia y Santa Catalina	198	180		

**Table 2.** Ten Colombian MNs with highest number of bird species recorded in this paper.

**DP**	**MN**	**Number of bird species**
Cauca	Santa Rosa	1033
Putumayo	Mocoa	1002
Nariño	Ipiales	1000
Nariño	Córdoba	993
Nariño	Puerres	992
Nariño	Potosí	991
Putumayo	San francisco	983
Caquetá	San Vicente del Caguán	949
Caquetá	Florencia	928
Caquetá	El paujil	911

**Table 3.** Number of bird species per CAR in Colombia obtained in this paper.

**CAR**	**Number of bird species this work**
Corporación Autónoma Regional del Cauca	1409
Corporación Autónoma Regional de Nariño	1384
Corporación para el Desarrollo Sostenible del Sur de la Amazonia	1199
Corporación Autónoma Regional de la Orinoquía	1106
Corporación para el Desarrollo Sostenible del Área de Manejo Especial La Macarena	1065
Corporación Autónoma Regional para el Desarrollo Sostenible del Chocó	1059
Corporación para el Desarrollo Sostenible de Urabá	1041
Corporación Autónoma Regional del Valle del Cauca	983
Corporación Autónoma Regional del Centro de Antioquia	963
Corporación Autónoma Regional de la Frontera Nororiental	940
Corporación Autónoma Regional de los Valles del Sinú y San Jorge	928
Corporación Autónoma Regional del Guavio	924
Corporación Autónoma Regional de Chivor	896
Corporación Autónoma Regional de Boyacá	893
Corporación Autónoma Regional del Cesar	882
Corporación Autónoma Regional de las cuencas de los ríos Rionegro y Nare	862
Corporación Autónoma Regional de Caldas	861
Corporación Autónoma Regional de Santander	861
Corporación Autónoma Regional de Cundinamarca	835
Corporación Autónoma Regional para la Defensa de la Meseta de Bucaramanga	824
Corporación Autónoma Regional del Tolima	821
Corporación Autónoma Regional de la Guajira	770
Corporación Autónoma Regional del Alto Magdalena	748
Corporación para el Desarrollo Sostenible del Norte y Oriente de la Amazonia	725
Corporación Autónoma Regional de Risaralda	719
Corporación Autónoma Regional del Magdalena	697
Corporación Autónoma Regional del Sur de Bolívar	636
Corporación Autónoma Regional del Quindío	633
Área Metropolitana del Valle de Aburrá	510
Corporación Autónoma Regional del Atlántico	510
Corporación Autónoma Regional del Canal del Dique	506
Corporación Autónoma Regional de Sucre	500
Departamento Administrativo Distrital del Medio Ambiente de Santa Marta	488
Establecimiento Público Ambiental	486
Departamento Técnico Administrativo del Medio Ambiente Barranquilla	482
Corporación para el Desarrollo Sostenible de la Mojana y del San Jorge	479
Secretaria Distrital de Ambiente de Bogotá D.C.	378
Departamento Administrativo de Gestión del Medio Ambiente de Santiago de Cali.	308
Corporación para el Desarrollo Sostenible del Archipiélago de San Andrés Providencia y Santa Catalina	198

**Table 4.** Bird species per PAs in Colombia. Classification of PAs used here is: Parque Nacional Natural (PNN), Santuario de Flora (SF), Santuario de Fauna (SFA), Reserva Nacional Natural (RNN), Vía Parque (VP), Santuario de Flora y Fauna (SFF) and Área Natural Única (ANU).

**Category**	**PA**	**Number of bird species this work**	**Number of bird species in other sources**	**References of other sources**
PNN	Sumapaz	919		
PNN	Chingaza	911	531	Linares-Romero et al. 2020
PNN	Serranía de los Churumbelos	909	421	Salaman et al. 1999
PNN	Alto Fragua Indiwasi	893		
PNN	Cordillera de los Picachos	877		
SF	Plantas Medicinales Orito Ingi Ande	860		
PNN	Paramillo	846		
PNN	Los Farallones de Cali	840		
PNN	El Cocuy	787		
PNN	Serranía de los Yariguíes	775	583	Donegan et al. 2010
PNN	Tama	737		
PNN	Las orquídeas	729		
PNN	Munchique	711		
PNN	Sierra de la Macarena	697	183	Rangel-Ch. et al. 1995a
PNN	Serranía de Chiribiquete	465	355	Álvarez et al. 2003
PNN	Tinigua	628	441	Cadena et al. 2000
PNN	Sierra Nevada de Santa Marta	616		
PNN	Amacayacu	615	355	Rangel-Ch. 1995
PNN	Los Katíos	609		
PNN	La Paya	596		
PNN	Cahuinarí	592		
PNN	Complejo volcánico Dona Juana Cascabel	586		
PNN	Rio Pure	570		
PNN	Yaigojé Apaporis	562		
SFA	Acandí Playón	541		
RN	Nukak	539		
RN	Puinawai	516		
PNN	Utría	504		
VP	Isla de Salamanca	498		
PNN	Nevado del Huila	493		
PNN	Los Nevados	489	162	Rangel-Ch. and Garzón-C. 1995
PNN	Tayrona	485	200	Rangel-Ch. and Lowy-C. 1995
SFF	El Corchal “El Mono Hernández”	484		
PNN	El Tuparro	482	320	Rangel-Ch. et al. 1995b
PNN	Puracé	477		
PNN	Sanquianga	477		
PNN	Tatamá	467		
SFF	Los Flamencos	465		
PNN	Catatumbo Bari	457		
PNN	Los Corales del Rosario y San Bernardo	457	141	Duque-García and Franke-Ante 2011
PNN	Uramba Bahía Málaga	456		
SFF	Ciénaga Grande de Santa Marta	453		
PNN	Las Hermosas	440		
PNN	Pisba	429		
SFF	Los Colorados	420		
PNN	Selva de Florencia	419	357	Gómez et al. 2020
SFF	Isla de la Corota	304		
PNN	Cueva de los Guacharos	393		
SFF	Galeras	349		
ANU	Los Estoraques	342		
SFF	Otún Quimbaya	340		
SFF	Guanentá Alto Rio Fonce	330		
SFF	Iguaque	322		
PNN	Bahía Portete-Kaurrele	277		
PNN	Macuira	250		
PNN	Old Providence Mc Bean Lagoon	194		
PNN	Gorgona	84		
PNN	Corales de Profundidad	39		

**Table 5.** Ten Colombian CTTs with highest number of bird species recorded in this paper. Territories included here are indigenous reservations and afro-descendant communities.

**Category**	**Collective Titling Territory**	**Ethnic group or community**	**Number of bird species**
Indigenous reservation	Kamëntšá Biÿá de Sibundoy	Kamëntšá	993
Afro-descendant communities	La Nueva Esperanza	La Nueva Esperanza	961
Indigenous reservation	Nasa Uh	Nasa	946
Indigenous reservation	Yunguillo	Inga	876
Indigenous reservation	Simorna	Emberá Chamí	875
Indigenous reservation	La Florida	Paéz	869
Indigenous reservation	Alto Orito	Emberá Chamí	865
Indigenous reservation	Inga de Condagua	Inga	862
Indigenous reservation	San José	Inga	861
Indigenous reservation	Rumiyaco	Pasto	856
